# Tracheostomy among Children Admitted in the Pediatric Intensive Care Unit of a Tertiary Care Centre

**DOI:** 10.31729/jnma.8323

**Published:** 2023-11-30

**Authors:** Rahul Bathwal, Kripa Dongol, Heempali Dutta, Yogesh Neupane

**Affiliations:** 1 Department of ENT-HNS, Tribhuvan University Teaching Hospital, Maharajgunj, Kathmandu, Nepal

**Keywords:** *mechanical ventilation*, *complication*, *pediatric*, *tracheostomy*

## Abstract

**Introduction:**

Tracheostomy is commonly performed for upper airway obstruction, prolonged mechanical ventilation and tracheo-bronchial toileting. Pediatric tracheostomy differs from adult tracheostomy in terms of surgical procedure, post-operative care and recovery. The tracheostomized patients may either be decannulated, discharged with tube-in-situ or the patient may expire. The aim of this study was to find out the prevalence of tracheostomy in patients admitted to the Pediatric intensive care unit of a tertiary care centre.

**Methods:**

A descriptive cross-sectional study was performed among children admitted to the Pediatric intensive care unit of a tertiary care centre from 1 May 2017 to 31 August 2022 after obtaining ethical approval from the Institutional Review Committee. A convenience sampling method was used. The point estimate was calculated at 95% Confidence Interval.

**Results:**

Among 1472 patients, tracheostomy was done in 65 (4.41%) (3.37-5.47, 95% Confidence Interval). A total of 33 (50.76%) underwent tracheostomy for prolonged ventilation whereas 32 (49.23%) were tracheostomized for airway obstruction. Among them, 41 (63.07%) patients were successfully decannulated, 9 (13.84%) were discharged with tracheostomy tubes in situ whereas 15 (23.07%) patients deceased. The most common complication was tracheostomy tube blockage reported in 5 (7.69%).

**Conclusions:**

The prevalence of tracheostomy among the children of the pediatric intensive care unit was found to be lower than in other studies.

## INTRODUCTION

Tracheostomy is the fashioning of a permanent opening between the trachea and the skin.^1^ It is commonly done for upper airway obstruction, prolonged mechanical ventilation and tracheobronchial toileting.^2^ Nowadays, due to the treatment of critically ill patients on mechanical ventilators, the rate of tracheostomy has increased.^3^

Pediatric tracheostomy is challenging in terms of surgical procedures as well as post-operative management.^4^ Once tracheostomized, either the patient could be successfully decannulated, discharged with a tracheostomy tube in situ for home-based care or the patient may expire. However, data on the outcome of tracheostomized patients is limited in our country, especially of the pediatric population. Data on the outcome of tracheostomy would help us to improve patient care and counsel the guardians regarding patient prognosis.

The objective of this study was to find out the prevalence of tracheostomy in patients admitted to the Pediatric intensive care unit (PICU) of a tertiary care centre.

## METHODS

This descriptive cross-sectional study was conducted among children admitted to the Department of PICU of Tribhuvan University Teaching Hospital, Maharajgunj, Kathmandu, Nepal from 1 May 2017 to 31 August 2022 after obtaining ethical approval from the Institutional Review Committee (Reference number: 163(6-11) E2 079/080) and data was collected from 25 September 2022 to 25 October 2022. All the children who were admitted to the PICU with complete hospital data were included in our study and patients with incomplete/missing data were excluded from the study. A convenience sampling method was used. The sample size was calculated using the following formula:


$$\begin{array}{ll} \text{n} & ={\text{Z}}^{2}\times \frac{\text{p}\times \text{q}}{{\text{e}}^{\text{2}}}\\ & =1.96^{2}\times \frac{0.50\times 0.50}{0.03^{2}}\\ & =1068 \end{array}$$

Where,

n = minimum required sample size
Z = 1.96 at 95% Confidence Interval (CI)
p = prevalence taken as 50% for maximum sample size
q = 1-p
e = margin of error, 3%

The calculated sample size required was 1068. However, the final sample size taken for the study was 1472.

The indications for tracheostomy were broadly classified as airway obstruction and prolonged mechanical ventilation either due to neurological causes or respiratory causes. All the tracheostomies were performed by Otorhinolaryngologists and post-operative care was provided in PICU at least for the initial 48 hours post-surgery. The outcomes of tracheostomy were classified as decannulated (closure of stoma), discharged with tube-in-situ and deceased (death due to primary disease or death due to tracheostomy-related cause). Complications of tracheostomy were also recorded.

Data was entered into Microsoft Excel 2007 and analysed using IBM SPSS Statistics version 20.0. The point estimate was calculated at a 95% CI.

## RESULTS

Among 1472 patients, the prevalence of tracheostomy was 65 (4.41%) (3.37-5.47, 95% CI). Out of them, 45 (69.23%) were male and 20 (30.76%) were female. The age of patients ranged from newborn to 14 years, with a median age of 4 years. A total of 33 (50.76%) patients underwent tracheostomy for prolonged ventilation whereas 32 (49.23%) patients were tracheostomized for airway obstruction (Table 1).

**Table 1:** Various indications of tracheostomy (n=65).

Indications	n (%)
Airway obstruction	32 (49.23)
Prolonged intubation
Neurological cause	25 (38.46)
Respiratory cause	8 (12.31)

A total of 41 (63.07%) of patients were successfully decannulated. There were 9 (13.84%) patients who were discharged with tracheostomy tubes in situ whereas 15 (23.07%) were deceased (Table 2).

**Table 2:** Outcomes of patients according to indications of tracheostomy (n= 65).

Outcomes	n (%)
Decannulated	41 (63.07)
Tracheostomy tube in situ	9 (13.84)
Deceased	15 (23.07)

There was a total of 10 (15.38%) complications related to tracheostomy. The most common complication was tracheostomy tube blockage seen in 5 (7.69%) (Table 3).

**Table 3:** Complications of tracheostomy (n= 65).

Complications	n (%)
Tube blockage	5 (7.69)
Tube dislodgement	2 (3.08)
Surgical emphysema	1 (1.54)
Suprastomal granulations	1 (1.54)
Trachea-cutaneous fistula	1 (1.54)

## DISCUSSION

The prevalence of pediatric tracheostomy among children admitted to the PICU was found to be 4.41% which is lower than studies done in similar settings.^5^ Forty-one (63.07%) patients were successfully decannulated after tracheostomy. Nine (13.84%) patients were discharged with tracheostomy tube in situ. Fifteen (23.07%) patients expired either due to primary pathology or due to tube-related complications.

The rate of pediatric tracheostomy for acute respiratory obstruction has declined due to immunization against Haemophilus influenzae, Corynebacterium diphtheriae and Streptococcus pneumoniae.^6,7^ Furthermore, tracheostomy for such infective conditions has declined because these conditions are frequently managed with endotracheal intubation.^7^ However, the overall rate of tracheostomy has increased due to the treatment of patients under mechanical ventilation.^3^ In our study, nearly half of the patients were tracheostomized for prolonged ventilation while the other half were tracheostomized for upper airway obstruction.

The decannulation rate of tracheostomy varied in different studies from 8.8% to 42%.^8^ A study has shown a decannulation rate of 39.1%,^8^ 38%.^9^ In our study, 41 (63.07%) patients could be decannulated which is similar to that of a study where a decannulation rate of 62.7% and 60% respectively was found.^10,11^ The decision for safe decannulation may be difficult in pediatric tracheostomy cases due to the possibility of adverse outcomes.^12^ In our study, patients who recovered from their primary respiratory and neurological pathology were decannulated in the majority of cases. However, patients with failed airway procedures or those with multi-level airway obstruction are still on tracheostomy tubes. Patients tracheostomized for trauma are more likely to be decannulated than those with congenital or chronic pathology.^8,12^ In our study, all six patients who had head injuries were decannulated.

Twenty patients (62.50%) with tracheostomy for airway obstruction were decanulated in our study. A study has shown a 74.8% decannulation rate in patients with laryngotracheal obstruction.^10^ This difference may be due to variations in patient characteristics and surgical expertise. In Nepal, the management of complex airway cases are mostly referred to our department, therefore, airway cases form the majority of indication for tracheostomy in this study. Patients with bilateral abductor palsy, subglottic stenosis, glottic web, true vocal cord cyst and foreign body in the airway underwent different surgical procedures, following which decannulation was possible in the majority. These patients required multiple surgeries and were tracheostomized for a relatively longer duration than those patients who were tracheostomized for prolonged intubation.

In our study, patients with supraglottic stenosis and laryngeal trauma are still tracheostomized due to failed airway procedures. Patients with congenital problems such as Pyriform aperture stenosis, Pierre-Robin Sequence and Tracheo-oesophageal fistula had mortality in the postoperative period due to tube blockage. All three patients were below one year of age. Therefore, infants, especially those having congenital malformations seem to be at a higher risk of tube-related mortality. A study also reported that an age of less than 1 year is a risk factor for mortality.^7^ In contrast, 56.3% of patients with craniofacial anomalies were decannulated in another study.^10^ These patients required a longer duration of tube placement and multiple airway surgeries.

Pediatric tracheostomy is associated with higher mortality as compared to adults due to various degrees of medical complexity.^6^ The mortality of pediatric tracheostomy ranges from 8 to 20%.^2,13^ Similarly, tracheostomy-specific mortality ranges from 0 to 3.5%.^2,6^ Another study has reported 23% overall mortality while few studies have reported 18% mortality.^9,14^ A study found 27% tube-related mortality.^15^ In our study, the overall mortality rate of tracheostomized patients was 23.07% and the tracheostomy-specific death was 9.23%. These variations could be due to different ages and indications of tracheostomy, pre-existing comorbidities and post-operative care. A study reported poorer prognosis in patients tracheostomized for neurological diseases.^9^ Some studies reported that patients tracheostomized for cardiopulmonary disease and bronchopulmonary dysplasia had a higher mortality rate than those tracheostomized for airway obstruction.^2,10^ Improved survival has been reported in patients tracheostomized in hospitals having a high caseload for pediatric tracheostomy.^7^ This may be due to better surgical expertise and care being provided by experienced nursing staff.

In this study, the most common cause of tracheostomy-related death was tube blockage and tube dislodgement. Two patients had tube dislodgement and both of them expired. Out of five patients with tube blockage, four expired while one patient revived. We found that these tube-related mortality were during the initial two years of this study which drastically declined as the expertise of surgeons increased and as nurses received training for tracheostomy care. Tube blockage and accidental decannulation were the most dreadful adverse events. These complications are potentially avoidable with high-standard post-operative care.

The overall complication rate in this study was 15.38% which is comparable to a study which showed a complication rate of 20.1%.^8^ Patient with suprastomal granulations was decannulated after its excision. A patient with surgical emphysema had tube dislodgement as well and that patient expired. The patient with a trachea-cutaneous fistula underwent its closure and was decannulated.

The main limitation of this study was its retrospective nature. It would have been better if we had calculated the median duration for decannulation, average time of complication or tube-related death. Further study may be performed to see whether early or delayed tracheostomy affects the outcome in ventilated patients.

## CONCLUSIONS

The prevalence of pediatric tracheostomy among the children admitted to PICU was found to be lower than in other studies done in similar settings. Common causes for tube-specific mortality were tube obstruction and dislodgement. Infants with craniofacial abnormalities had relatively higher tube-specific mortality.
